# Ultrasound-targeted microbubble destruction mediated miR-492 inhibitor suppresses the tumorigenesis in non-small cell lung cancer

**DOI:** 10.1080/07853890.2021.2005254

**Published:** 2021-11-24

**Authors:** Wendi Zou, Yan Wang, Qingqing Song, Qianqian Li, Jie Ren, Xiaoyu Liu, Wei Cui

**Affiliations:** Ultrasound Department, Shengli Oilfield Central Hospital, Dongying, China

**Keywords:** MicroRNA-492, non-small cell lung cancer, proliferation, migration, invasion, ultrasound-targeted microbubble destruction

## Abstract

**Background:**

Ultrasound-targeted microbubble destruction (UTMD) is a novel adjuvant tumor therapeutic method by enhancing exogenous gene transfection to target tissues. This study aims to investigate the role of microRNA-492 (miR-492) in non-small cell lung cancer (NSCLC) and further analyze the effects of UTMD-mediated miR-492 inhibitor on tumorigenesis.

**Methods:**

The expression of miR-492 was detected by qRT-PCR. Co-transfection of microbubbles and miR-492 inhibitor with Lipofectamine 3000 was performed to achieve UTMD-mediated miR-492 inhibition in NSCLC cells. CCK-8 and Transwell assay were used to determine NSCLC cell proliferation, and the migration and invasion.

**Result:**

High expression of miR-492 was associated with poor prognosis in NSCLC patients. miR-492 inhibitor suppressed tumor cell proliferation, migration and invasion, and UTMD not only increased the transfection efficiency of miR-492 inhibitor, but also enhance the inhibitory effects on cell biological behaviors.

**Conclusion:**

The results showed that the expression level of miR-492 was up-regulated in NSCLC tissue samples and cells. Silencing of miR-492 inhibited NSCLC cell proliferation, migration and invasion, and UTMD-mediated miR-492 inhibitor could promote more significant inhibition, which indicated that UTMD-mediated miR-492 inhibitor might provide a novel strategy for the treatment of NSCLC.

**KEY MESSAGES**
- miR-492 inhibitor inhibited cell proliferation, migration and invasion.
- UTMD-mediated miR-492 inhibitor can promote more significant inhibition.
- UTMD-mediated miR-492 inhibitor provide a new strategy for NSCLC.

## Introduction

Lung cancer is the leading cause of cancer-related death worldwide [1]. Lung cancer is traditionally classified into small cell lung cancer and non‐small cell lung cancer (NSCLC) [2]. NSCLC is the most common type of lung cancer, accounting for approximately 80–90% of all lung cancers [3], and has a slow onset and slow spread of cancer relative to small cell lung cancer. However, about 75% of patients are found to be in middle or advanced stages at the initial diagnosis, which leads to a great burden for disease control and treatment. Approximately 20–25% of the patients were candidates for surgical resection [4]. A high proportion of patients with resected NSCLC die of recurrent NSCLC, and the 5-year survival rate was very low [5]. The discovery of targetable oncogenic mutations revolutionized treatment choices for NSCLC, yet biomarker targeted therapies for NSCLC are still evolving.

Ultrasound-targeted microbubble destruction (UTMD) technology is a non-viral, novel approach to gene therapy, in which plasmid DNA is targeted to specific tissues and organs *in vivo* [6]. The delivery of genes by intravenously injecting plasmid DNA encapsulated in microbubbles into the animal’s bloodstream, to lyse them selectively in the microcirculation of specific organs via ultrasound, is highly innovative [7,8]. The synergistic effect of UTMD in targeted trafficking has been reported by many studies [9–11]. For instance, Wang et al. considered that UTMD enhanced the synergistic treatment of rheumatoid arthritis via targeting liposomes [9]. Additively, research revealed that UTMD-mediated miR-767 inhibition resulted in a more significant suppressive effect on NSCLC tumour proliferation, migration and invasion [10]. Moreover, in other cancers or malignancies, such as hepatocellular carcinoma [11], synergistic therapeutic effects of UTMD were also widely recognized. Consequently, UTMD has been considered a promising adjunct to targeted therapy for human malignancies.

MicroRNAs (miRNAs) are a class of short (with an average of 22 nucleotides) endogenously initiated noncoding RNAs that have crucial roles in cancer development and progression [12]. Research evidence suggested that miRNAs could be delivered that target the tumour in passive, active, and stimuli-responsive ways [13], thus significantly affecting the course of tumour development. microRNA-492 (miR-492), as a short non-coding RNA, has also been reported to be aberrantly expressed in a variety of diseases including cancers and played an important role in disease progression [14–17]. miR-492, derived from the keratin 19 gene, was first mentioned to be upregulated in metastatic hepatoblastoma in research of Frowein et al. [14]. And, serum levels of miR-492 were significantly increased in the acute phase of acute myocardial infarction (AMI), implying that miR-492 also played an important clinical role in the diagnosis of AMI [15]. Moreover, in cancer-related areas, the aberrant expression and tumour-promoting activity of miR-492 have been reported in bladder cancer [16]. Other works of research have also demonstrated that low expression of miR-492 leads to inhibited SOCS2 expression, ultimately inhibiting the proliferation and migration of PCa cells [17]. However, the understanding of the relationship between miR-492 and NSCLC remains limited. Several miRNAs have also been analyzed in the development and progression of NSCLC [18]. For example, miRNA-148a is significantly correlated with tumour progression and poor clinical outcomes in lung cancer and can inhibit the migration and invasion of tumour cells [19]. Considering the important guiding function of miRNAs in cancer therapy [20], it is necessary to further explore the biological function of miR-492 in NSCLC.

In this study, the expression of miR-492 in NSCLC tissues was assessed, and the biological function of miR-492 was explored by *in vitro* experiments. In addition, based on the pre-existing study data of UTMD, this study aimed to compare the differences in NSCLC cell biological behaviours under conventional miR-492 transfection or UTMD mediated miR-492 transfection. The results may provide a theoretical basis for the targeted therapy of UTMD mediated miRNAs.

## Methods

### NSCLC patients and tissue collection

Tumour tissues involved in this study were collected from 96 NSCLC patients who were confirmed by histopathological examination between April 2017 and March 2020 in Shengli Oilfield Central Hospital. Cancer tissues and adjacent non-cancerous tissues were collected from all patients by surgical resection, then were frozen in liquid nitrogen for further use. The clinicopathological characteristics of all patients were recorded throughout the treatment course for subsequent analysis. Patients who received preoperative treatment were excluded from our study. The protocols for tissue collection and analysis were all in accordance with the guideline of the Ethics Committee of Shengli Oilfield Central Hospital and has approved by the Ethics Committee of Shengli Oilfield Central Hospital. Signed informed consent was obtained from the patients (or guardians) before sampling.

### Cell lines and cell culture

Four NSCLC cell lines (A549, H460, PC9, H522) and a normal lung cell line (NHBE) were purchased from the Cell Bank of the Chinese Academy of Science (Shanghai, China) were used in this study. All cells were incubated in Dulbecco’s modified Eagle medium (DMEM, Invitrogen, Thermo Fisher Scientific, Inc., Waltham, Massachusetts) supplemented with 10% foetal bovine serum (FBS, Invitrogen), 100 U/ml penicillin and 100 mg/l streptomycin, in a saturated humidified atmosphere with 5% CO_2_ at 37 °C.

### Cell transfection

Cell transfection was used to achieve the regulation of miR-492 *in vitro* in this study. Inhibitor NC (5′-CAGUACUUUUGUGUAGUACAA-3′) and miR-492 inhibitor (5′-AAGAAUCUUGUCCCGCAGGUCCU-3′) were synthesised from Gene Pharmaceuticals (Shanghai, China) and transfected into A549 and PC9 cells using Lipofectamine 3000 (Invitrogen, California, USA). Cells transfected with transfection reagents alone were set as control. After 48 h of transfection, the cells were available for further analysis.

### Microbubble preparation and cell transfection

In the experiment, the microbubbles were obtained by ultrasonic treatment. The microbubbles were performed by sonication of 0.4 mg/ml 1,2-distearoyl-3-trimethylammoni-umpropane (Avanti Polar Lipids, Inc.) with 1 mg/ml polyethyleneglycol-2000 stearate (Avanti Polar Lipids, Inc.), 2 mg/ml distearoylphosphatidylcholine (Avanti Polar Lipids, Alabaster, Inc.) and perfluoropropane gas, the ultrasonic irradiation intensity was 0.5 W/cm^2^ and the irradiation time was 60 s. miR-492 inhibitor or inhibitor NC was incubated with the microbubbles for 30 min at 37 °C. The mixture was added to A549 and PC9 cells and transfected with Lipofectamine 3000 (Invitrogen; Thermo Fisher Scientific, Inc.), then ultrasound irradiation was applied with an irradiation intensity of 0.5 W/cm^2^.

### RNA extraction and quantitative reverse transcription PCR (qRT-PCR)

Total RNA was obtained from fresh tissue samples and cells by TRIzol reagent (Invitrogen, Life Technologies, Paisley, UK). The obtained RNA was reversed transcribed into single-stranded cDNA with PrimeScript reverse transcriptase kit (TaKaRa, Shiga, Japan) following the manufacturer’s guidelines. The expression level of miR-492 was determined by qRT-PCR with the SYBR green I Master Mix kit (Invitrogen, Carlsbad, California, USA) on a 7500 real-time polymerase chain reaction system (Applied Biosystems, USA). U6 was used as an endogenous control for miR-492. The sequences were as follows: miR-492 (F): 5′-GCCGAGAGGACCTGCGGGA-3′, miR-492 (R): 5′-CTCAACTGGTGTCGTGGA-3′; U6 (F): 5′-CTCGCTTCGGCAGCACA-3′, U6 (R): 5′-AACGCTTCACGAATTTGCGT-3′. The final expression value was calculated using the 2^−ΔΔCt^ method.

### Cell proliferation assay

The effect of miR-492 on the proliferation of A549 and PC9 cells after transfection was detected by cell counting kit 8 (CCK-8). Cells were seeded into a 96-well plate (2 × 10^3^ cells/well) and incubated for 0, 24, 48 and 72 h. Then, 10 μl of CCK-8 reagent (Beyotime, Shanghai, China) was added to each well and further incubated for 2 h. The microplate reader was used to read the optical density at 450 nm.

### Analysis of cell migration and invasion

Transwell chambers (Corning, NY, USA) were applied for the measurement of cell migration and invasion of NSCLC cells in this study. Transwell chambers precoated with Matrigel (Corning, NY, USA) were used for invasion assay, while the Transwell chambers without Matrigel coating were used for migration assay. The transfected cells with a density of 2 × 10^5^ cells/well, seeded into a chamber. The upper chambers with serum-free medium and the low chambers were filled with culture medium supplemented with 10% FBS as a chemoattractant. The cells in the lower chambers were stained after 48 h of incubation and observed for counting under an inverted microscope (Olympus Corporation, Tokyo, Japan).

### Statistical analysis

All quantitative data were expressed as mean ± standard deviation (SD) and analyzed at SPSS 21.0 (SPSS, Inc., Chicago, Illinois) and GraphPad 7.0 (GraphPad Software, Inc., USA). The student’s *t*-test was used to compare the differences between two groups, and one-way ANOVA followed by Tukey’s test was used for differences between multiple groups. The comparison between miR-492 expression and clinicopathological characteristics was performed by the Chi-square test. The included NSCLC patients were followed up to record their survival information, and Kaplan–Meier survival curves were plotted for patients with different miR-492 expression levels, and a log-rank test was used to compare the different distributions between curves. *p* < .05 was considered to indicate a statistically significant difference.

## Results

### Upregulated expression of miR-492 in NSCLC patients and cells

**Figure 1.**
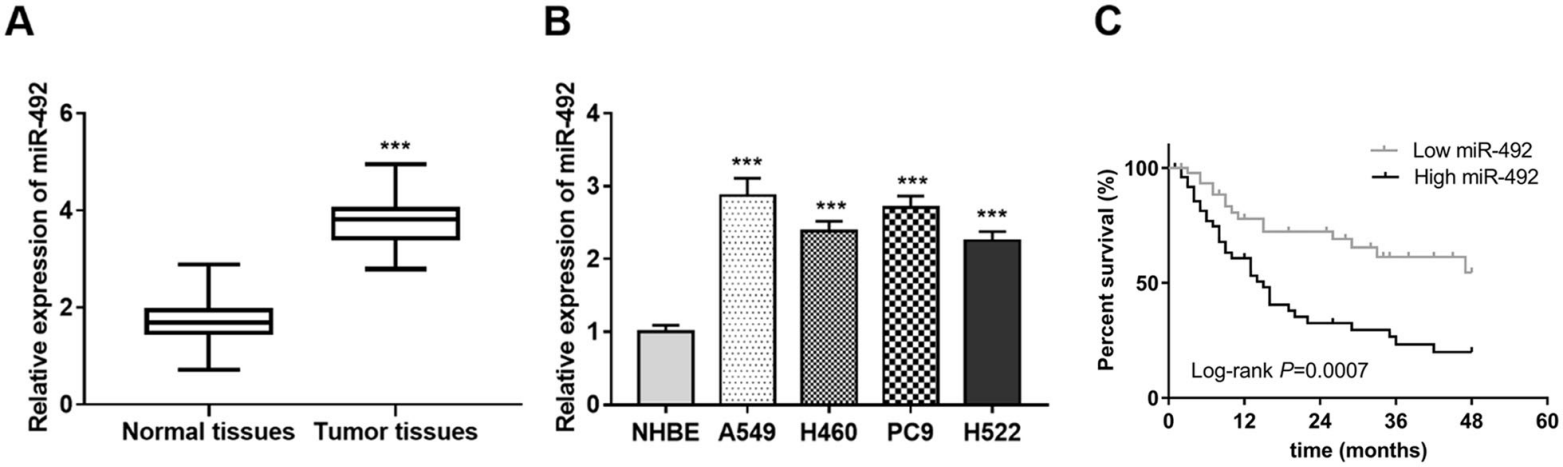
Expression of miR-492 in NSCLC tissues and cell lines. (A) The expression of miR-492 was increased in NSCLC tissues. (B) The expression level of miR-492 was increased in four NSCLC cell lines compare with normal NHBE cell lines. (C) NSCLC patients with high miR-492 expression had worse survival compared with low miR-492 expression, illustrating that NSCLC patients with high miR-492expression were associated with worse prognostic survival (Log-rank *p* = .0007) (***p < .001).

The results showed that the expression of miR-492 in NSCLC was significantly up-regulated compared with that in the normal control group (*p* < .001, Figure 1(A)). The expression levels of miR-492 in four NSCLC cell lines (A549, H460, PC9 and H522) were also observed increased (all *p* < .001, Figure 1(B)) compared with normal NHBE cell lines.

### Relationship between miR-492 and clinicopathological characteristics in patients with NSCLC

The role of miR-492 in the tumour development of NSCLC was examined by investigating the relationship between miR-492 and the clinicopathological data of cancer patients. According to the median value of the miR-492 expression, the NSCLC patients were grouped into low (*n* = 46) and high (*n* = 50) miR-492 expression groups. From the data in Table 1, the expression of miR-492 was found to be associated with tumour size (*p* = .039), TNM (tumour node metastasis) stage (*p* = .006) and lymph node metastasis (*p* = .002) whereas no significant relationship was found between miR-492 and age, gender, smoking, histological type or differentiation (all *p* > .05).

**Table 1.** Clinicopathological characteristics in patients with NSCLC.

Features	Total (*n* = 96)	miR-492		*p*-Value
		Low (*n* = 46)	High (*n* = 50)	
Age (years)				.963
≤60	29	14	15	
>60	67	32	35	
Gender				.629
Female	40	18	22	
Male	56	28	28	
Smoking				.936
No	33	16	17	
Yes	63	30	33	
Tumour size (cm)				.039
≤3	50	29	21	
>3	46	17	29	
Histological type				.497
Adenocarcinoma	55	28	27	
Squamous cell carcinoma	41	18	23	
Differentiation				.055
Well and moderate	55	31	24	
Poor	41	15	26	
TNM				.006
I–II	55	33	22	
III–IV	41	13	28	
Lymph node metastasis				.002
Negative	55	34	21	
Positive	41	12	29	

NSCLC: non-small cell lung cancer.

### Relationship between miR-492 and survival prognosis in patients with NSCLC

The follow-up survival information was assessed by constructing the Kaplan–Meier survival curves (Figure 1(C)), and the results showed that NSCLC patients with high miR-492 expression had worse survival compared with low miR-492 expression, illustrating that NSCLC patients with high miR-492 expression were associated with worse prognostic survival (Log-rank *p* = .0007). Furthermore, the relationship of miR-492 with NSCLC patients’ survival was adjusted by including the clinicopathological characteristics of patients into a multivariate Cox regression model. Analysis results revealed in Table 2 showed that TNM stage (*p* = .013), lymph node metastasis (*p* = .029), and miR-492 expression (*p* = .006) were independently associated with patients’ survival, and might be prognostic factors for NSCLC survival.

**Table 2.** Multivariate Cox regression analysis to adjust the association of miR-492 with NSCLC patients’ survival prognosis.

Variables	HR	95% CI	*p*-Value
Age	1.119	0.712–1.554	.423
Gender	1.208	0.769–1.637	.379
Smoking	1.375	0.894–1.981	.121
Tumour size	1.224	0.988–1.380	.096
Histological types	1.095	0.634–1.448	.557
Differentiation	1.427	0.976–1.909	.078
TNM stage	2.395	1.569–4.337	.013
Lymph node metastasis	1.875	1.347–2.888	.029
miR-492	2.994	1.815–4.894	.006

NSCLC: Non-small cell lung cancer; TNM: Tumour node metastasis.

### NSCLC cell proliferation, migration and invasion were inhibited by the knockdown of miR-492

**Figure 2.**
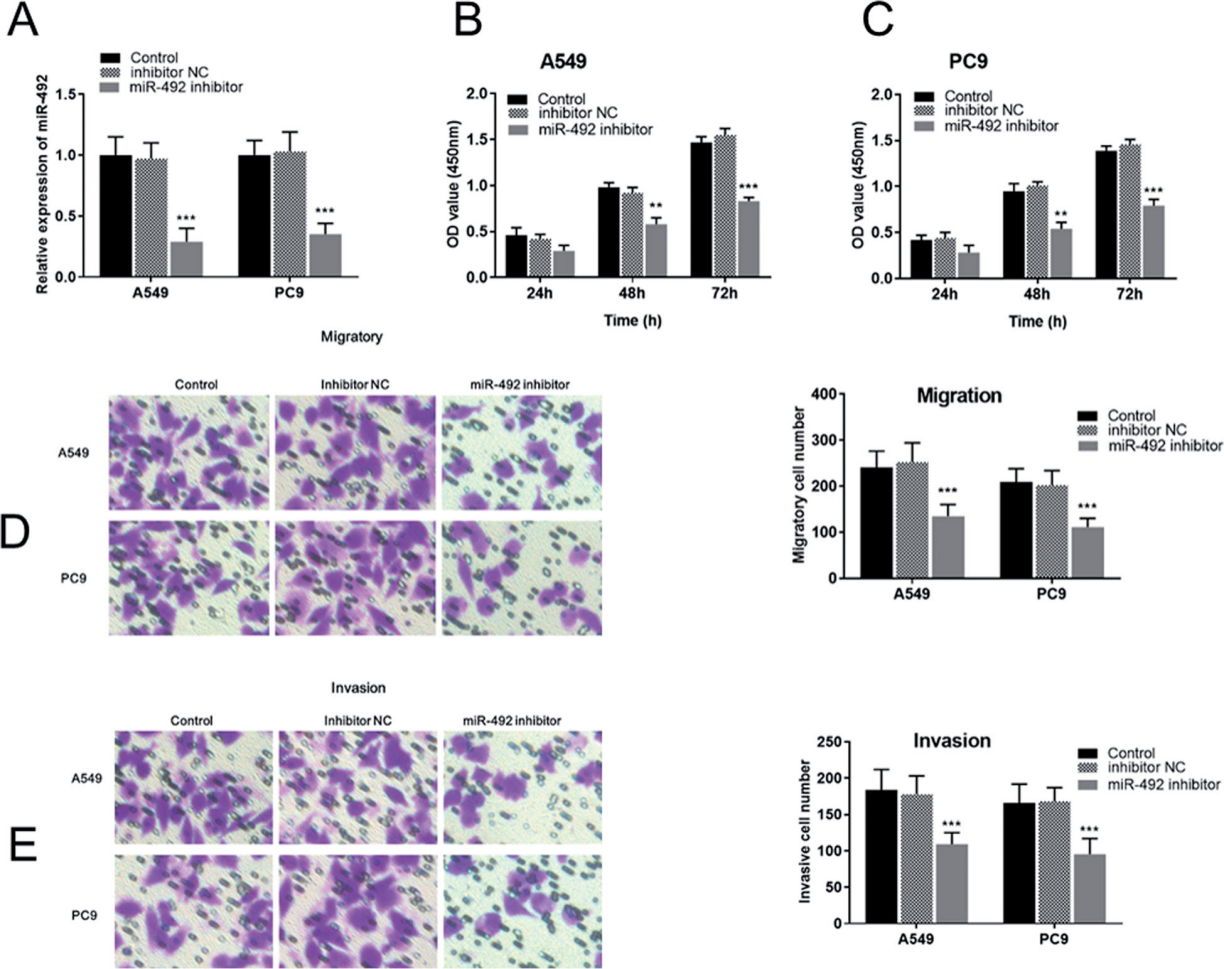
miR-492 inhibitor suppresses cell proliferation, migration and invasion of NSCLC cells. (A) After transfection with miR-492 inhibitors, miR-492 expression levels in A549 and PC9 cell lines decreased compared with inhibitor NC (all *p* < .001). (B, C) Silencing of miR-492 suppressed cell proliferation in A549 and PC9 cell lines compared with inhibitor NC, and the difference gradually became significant with time (48 h: all *p* < .01; 72 h: all *p* < .001). (D, E) Silencing of miR-492 significantly inhibited cell migration and invasion in A549 and PC9 cell lines compared with inhibitor NC. Transwell images observed by inverted microscopy showed that the number of cell migration and invasion was the least in A549 and pC9 cells transfected with miR-492 inhibitor (all *p* < .001) (**p < .01; ***p < .001).

Cellular experiments were performed in the study to analyze the function and role of miR-492 in the process of NSCLC. After transfection with miR-492 inhibitors, miR-492 expression levels in A549 and PC9 cell lines decreased compared with inhibitor NC (all *p* < .001; Figure 2(A)). Cell proliferation assays showed that silencing of miR-492 suppressed the cell proliferation of A549 and PC9 cell lines compared with controls, and the difference gradually became significant with time (48 h: all *p* < .01; 72 h: all *p* < .001; Figure 2(B,C)). Transwell assay was performed to evaluate the regulatory effect of miR-492 on cell migration and invasion in A549 and PC9 cells. The results showed that the silencing of miR-492 significantly inhibited the cell migration and invasion of A549 and PC9 cell lines compared with controls (all *p* < .001; Figure 2(D,E)). Besides, Transwell images observed by inverted microscopy showed that the number of cell migration and invasion was the least in A549 and pC9 cells transfected with miR-492 inhibitor (Figure 2(D,E)).

### UTMD significantly enhanced the transfection efficiency of miR-492 in NSCLC cells

**Figure 3.**
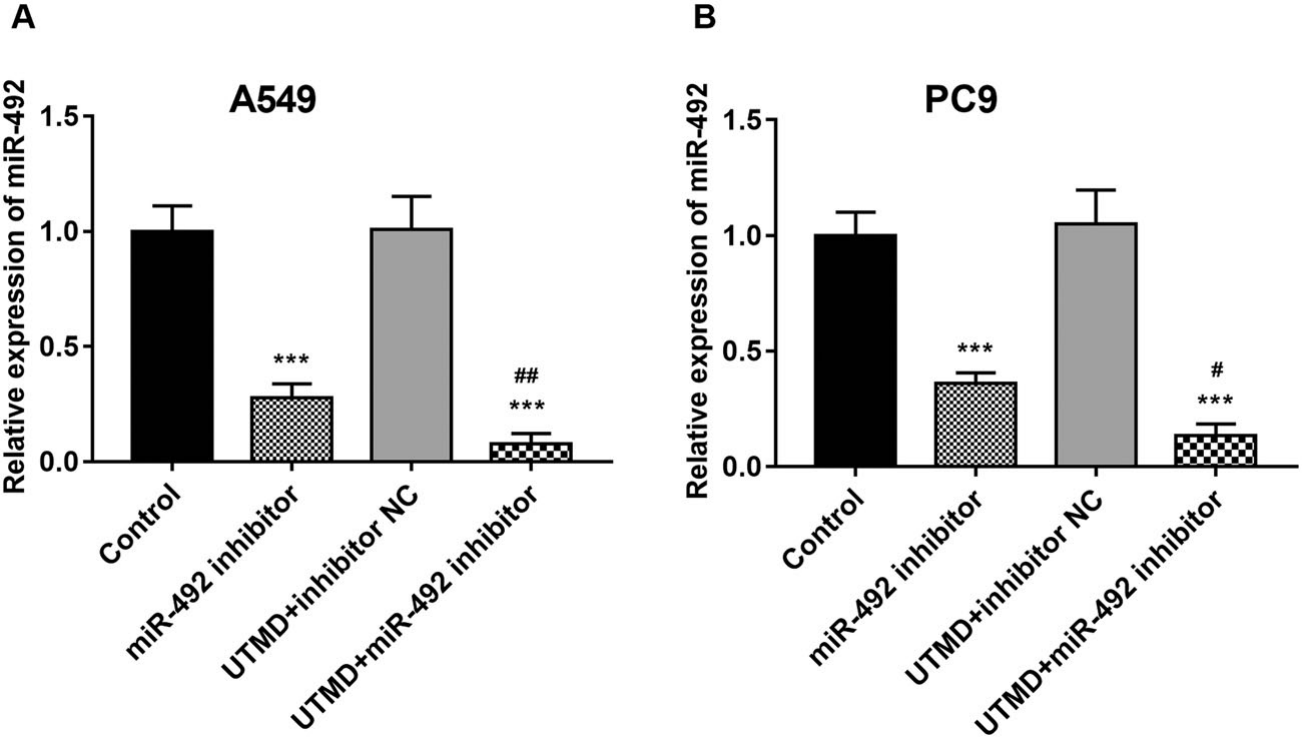
UTMD enhances cell transfection efficiency of miR-492. (A, B) UTMD could significantly enhance the inhibition of miR-492 inhibitor on the expression level of miR-4284 in A549 and PC9 cell lines, and the efficiency of transfection increased (***p < .001; ^#^*p* < .05; ^##^*p* < 0.01; *compare with control, ^##^compare with miR-492 inhibitor).

It was mentioned earlier that UTMD could enhance the transfection efficiency of exogenous genes into target tissues and organs. The effect of UTMD on miR-492 transfection efficiency was studied in our research. The results of the cell experiment showed that UTMD significantly enhanced the inhibitory effect of the miR-492 inhibitor on miR-492 expression in A549 and PC9 cells, which manifested by the further markedly decreased miR-492 expression induced by UTMD-mediated miR-492 inhibitor when compared with only miR-492 inhibitor (all *p* < .05; Figure 3).

### UTMD-mediated miR-492 inhibitor significantly suppressed NSCLC cell proliferation, migration and invasion

**Figure 4.**
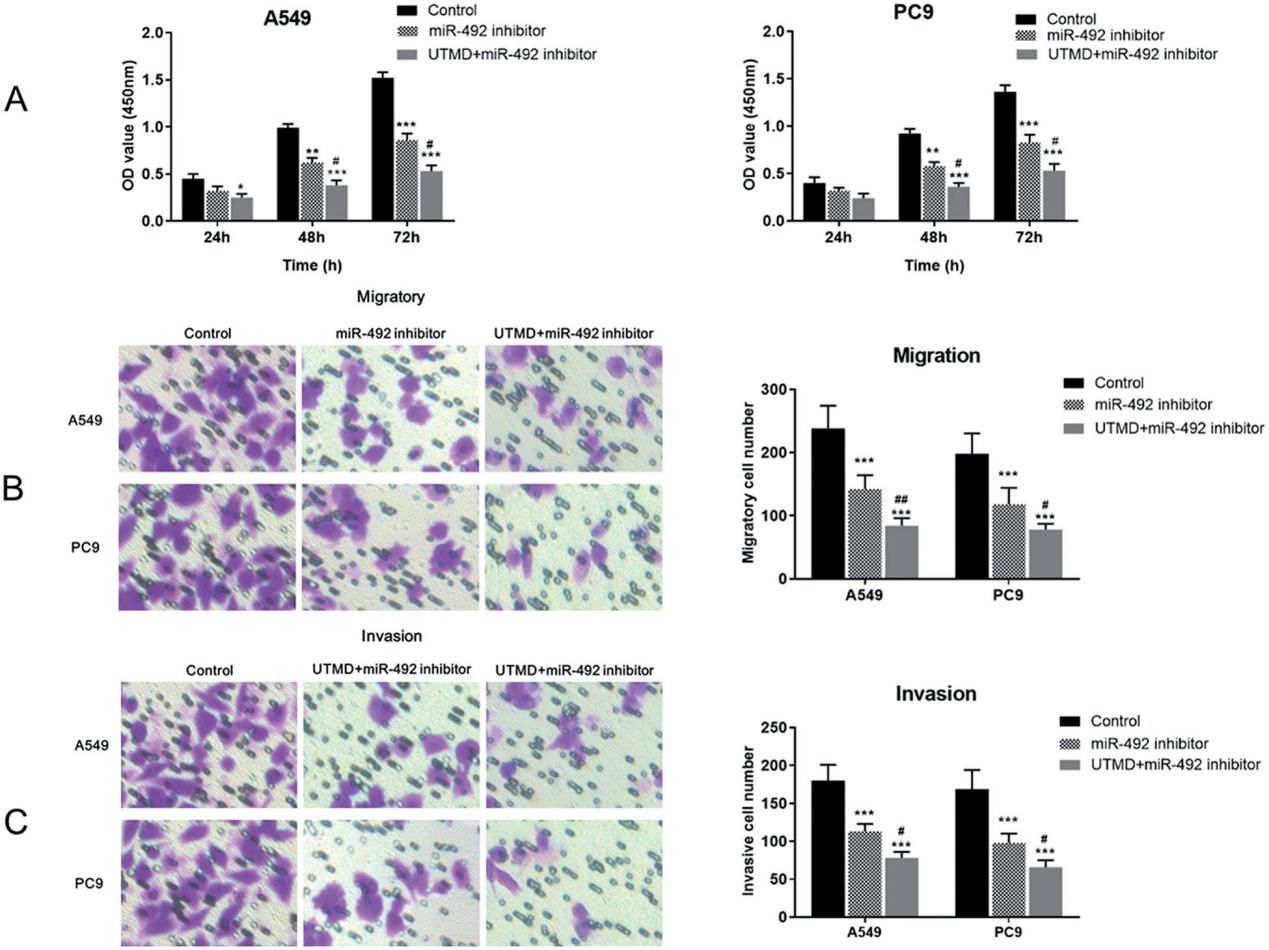
Effects of UTMD-mediated miR-492 inhibitor transfection on NSCLC cell proliferation, migration and invasion. (A) UTMD-mediated miR-492 inhibitor further inhibited cell proliferation compared with transfection of miR-492 inhibitor alone. (B, C) UTMD-mediated miR-492 inhibitor further inhibited cell migration and invasion compared with a single miR-492 inhibitor (*p < .05; **p < .01; ***p < .001; ^#^*p* < .05; *compare with control, ^#^compare with miR-492 inhibitor).

As shown in Figure 4(A), the UTMD-mediated knockout of miR-492 significantly inhibited the proliferation of A549 and PC9 cells compared with the control group. An enhanced inhibitory effect on cell proliferation was observed in cells co-transfected with UTMD and miR-492 inhibitor compared with cells transfected with miR-492 inhibitor alone (all *p* < .05). Further Transwell experiments showed that cell migration and invasion were significantly reduced after *in vitro* transfection with UTMD-mediated miR-492 inhibitor compared with the control group and the miR-492 inhibitor group (all *p* < .05; Figure 4(B,C)).

## Discussions

Lung cancer, that is, bronchogenic malignant tumours stemming from airway epithelioma, is the most often diagnosed cancer in the world and the most frequent cause of cancer death [21]. In 2012, approximately 1.6 million people died of lung cancer and it is estimated that the number of lung cancer deaths will increase to 3 million in 2035 [22,23]. NSCLC is the leading cause of malignancy-related mortality worldwide [24]. The discovery of targetable oncogenic mutations gives a more promising therapeutic prospect for the treatment of NSCLC. MicroRNAs (miRNAs) are a class of short (with an average of 22 nucleotides) endogenously initiated noncoding RNAs that have crucial roles in cancer development and progression [12]. Pidíkova et al. [25] reported that expression of miRNA clusters is decreased in colorectal cancer, and they show oncostatic capacity. Wang et al. [26] also published studies on lowly expressed miRNA-339-5p, which suppresses the malignant development of GC by negatively regulating ALKBH1. Kondrotienė et al. [27] revealed that miR-222 serves as a potential marker in distinguishing papillary thyroid carcinoma from nodular goitre in 2020. However, the biological function of miR-492 in NSCLC remains unclear and requires urgent further confirmation.

In this research, we found that miR-492 was abnormally expressed in NSCLC through data analysis. And previous studies showed that the expression level of miR-492 was altered in some diseases, indicating that miR-492 may be played a role in the development of diseases. In previous studies, Chang et al. [28] found that metapristone can be used for endometrial cancer treatment by regulating miR-492 and its new target genes Klf5 and Nrf1 *in vitro* and *in vivo*. Guo et al. [15] mentioned that the serum level of miR-492 remarkably increases in the acute phase of acute myocardial infarction, which may be used as an effective biomarker for diagnosing acute myocardial infarction. In this research, the results showed that the expression of miR-492 in NSCLC tissues was significantly up-regulated compared with normal control tissues. The expression levels of miR-492 were also observed to increase in the four NSCLC cell lines (A549, H460, PC9, H522) used in the experiment compared with normal NHBE cell lines. To further verify the accuracy, we selected A549 and PC9 whose expression levels were significantly upregulated and performed cell proliferation experiments and found that NSCLC cell proliferation ability decreased after the silencing of miR-492. Further cell migration and invasion assay results were more showing that cell migration and invasion ability were decreased when silencing of miR-492. Taken together, we hypothesized that miR-492 might be a tumour-promoting factor in NSCLC.

**Figure 5.**
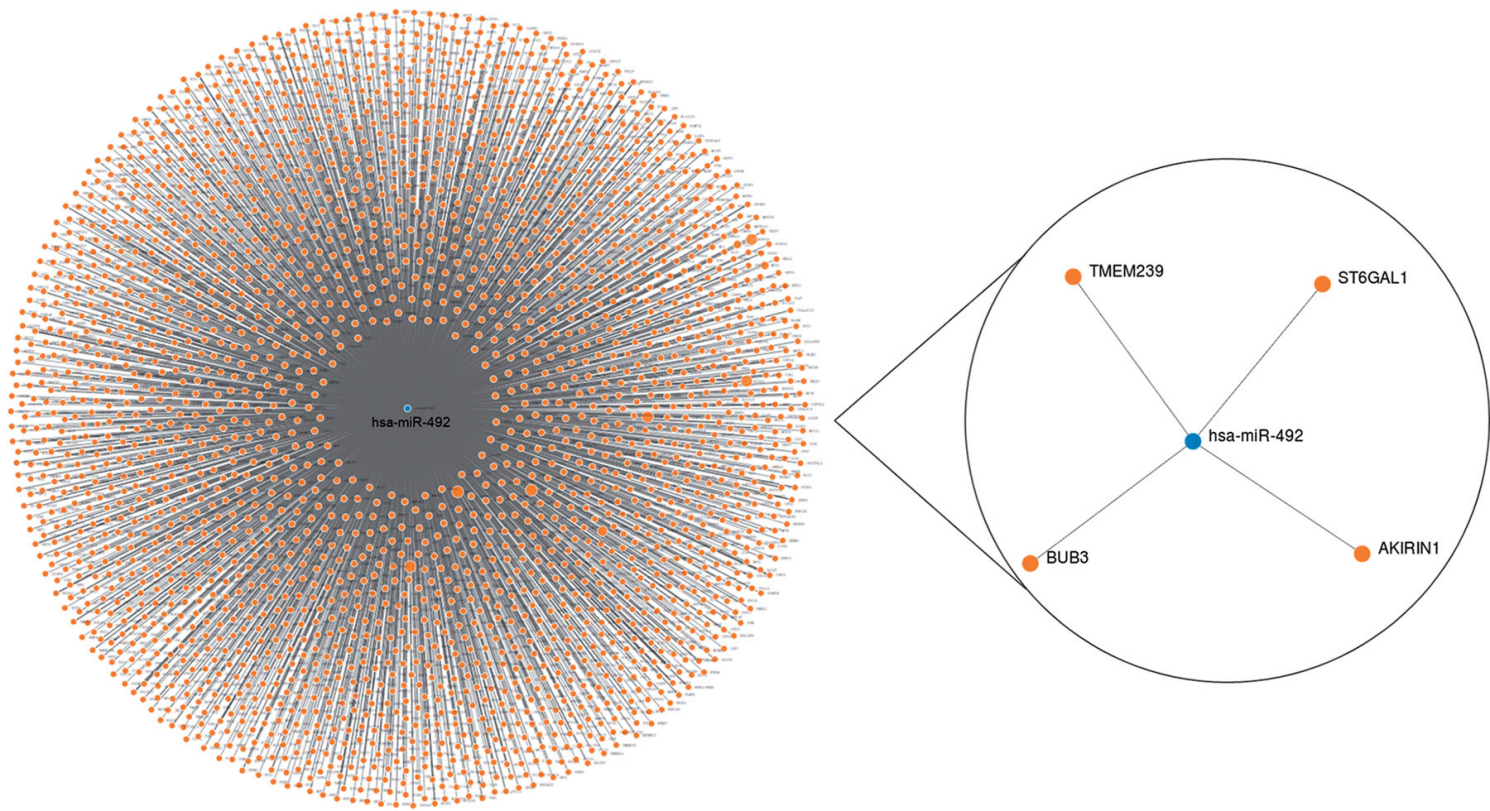
Target genes of miR-492 predicted by the miRWalk database.

Although we revealed that miR-492 might be a tumour-promoting factor in NSCLC progression, the specific mechanisms required further exploration. To preliminary investigate the potential target genes of miR-492, we used the miRWalk database to check the candidate genes that have complementary sequences of miR-492, and screened the potential targets using the data from three databases: TargetScan, miRDB and miRTarBase. Prediction results are shown in Figure 5, and TMEM239, ST6GAL1, BUB3 and AKIRIN1 were screened to be target genes for miR-492 according to the combined prediction results of the 3 databases. Among the screened target genes, the significant relationship of ST6GAL with tumour biomarkers has been reported and recognised by several studies [29]. A single-nucleotide polymorphisms research directly implicated that the ST6GAL1 gene was associated with lung carcinoma risk in the Chinese Han population [30]. For BUB3, Kang et al. found that BUB3 rs7897156C > T was associated with worse overall survival under a recessive model in NSCLC [31]. Regarding AKIRIN1, its critical role in immune responses and tumorigenesis was widely recognized [32]. Considering the critical roles of the predicted target genes in human malignancies, the relationship between miR-492 and the target genes needed to be explored is worth exploring in the future to enrich the mechanisms underlying the function of miR-492 in carcinogenesis.

Ultrasound-targeted microbubble destruction (UTMD) has been widely studied for gene therapy because of its low toxicity, low immunogenicity of vectors, minimum invasiveness, repeatability, and effectiveness [33,34]. As the current research focus of ultrasound molecular imaging, the ability of UTMD technology to enhance gene transfection efficiency has been confirmed by many findings. In the process of exosome drug delivery, Sun et al. [35] revealed that SonoVue™ microbubble together with UTMD significantly increases the infiltration and endocytosis of exosomes in these reluctant tissues, highlighting the potent potential of UTMD in facilitating exosomes delivery in tissues like the heart and adipose tissue. Lin et al. [36] reported that UTMD promoted the co-delivery of gemcitabine and miR-21, thereby improving the treatment of pancreatic cancer. Qin et al. [37] research demonstrated that cell proliferation, migration, and invasion were suppressed, and apoptosis was increased via using UTMD to successfully transfect PCa cells with miR-205 mimics plasmid. Similarly, UTMD also plays a great potential in non-small cell lung cancer treatment. For example, Li et al. [10] demonstrated the transfection efficiency of miR-767 inhibitors can be enhanced by UTMD, resulting in more significant suppression of the biological functions of NSCLC cells. In our study, through cellular experiments, we know that UTMD significantly enhanced the transfection efficiency of miR-492 in NSCLC cells and UTMD-mediated miR-492 inhibitor significantly suppressed NSCLC cell proliferation, migration, and invasion. This illustrates that UTMD enhanced the function of miR-492 inhibitor *in vitro*, thereby enhancing the inhibitory effect silencing of miR-492 on NSCLC tumour progression. This may provide useful evidence for the wider use of UTMD in NSCLC treatment.

Lipofectamine, which has been widely and commonly used for gene transfection into cultured cells [38]. The study by Shi et al. showed that a high level of transfection and transduction efficiency using Lipofectamine 3000 transfection reagent compared with Lipofectamine 2000 or FuGENE 6 reagents could be achieved [39]. In addition, studies have confirmed that Lipofectamine 3000™ had the least impact on cell morphology and viability [40]. Thus, UTMD and Lipofectamine 3000 (Invitrogen, California, USA) were combined in this study. Based on the cell experimental results, we speculated that both approaches may yield a synergistic utility such that cell transfection efficiencies WERE superimposed to significantly improve transfection efficiency. However, the efficiency of the UTMD technique was affected by the ultrasound irradiation conditions. Thus, the application of UTMD needs to be explored under conditions with different loading products and different cell strains.

The limitation of this study is that the results of this study are only *in vitro* experiments results. For the majority of patients, clinical treatments that bring about beneficial outcomes will be more convincing than research changes based on the molecular level, particularly this study was based on only preclinical or very limited clinical evidence. So further *in vivo* experiments or animal assays to confirm our conclusion and optimize the therapeutic effect are necessary. Our study used *in vitro* analyses to analyze the biological function of miR-492, as well as the enhanced functional role of UTMD-mediated miR-492 inhibitor in the progression of NSCLC. The analysis results provide evidence for miR-492 as an oncogene in NSCLC, and for the critical role of UTMD as an enhancer of miR-492 inhibitor in tumorigenesis. Further experiments, such as *in vivo* analysis, are necessary to confirm our conclusions in the future. Thus, we believe that the analysis results of our study will provide a theoretical and data basis for future *in vivo* and related experiments.

In conclusion, this study found that the expression of miR-492 was increased in NSCLC tissues and cells compared with the normal controls. miR-492 inhibitor can inhibit the proliferation, migration and invasion of tumour cells. After combining UTMD, UTMD can enhance the transfection efficiency of miR-492 inhibitor, and promote more significant inhibition of the biological function of NSCLC cells. It can be inferred from the above that miR-492 may be a potential therapeutic target for NSCLC, and UTMD-mediated delivery of miR-492 inhibitors may be a promising therapeutic strategy for NSCLC.

## Data Availability

The data used to support the findings of this study are available from the corresponding author upon reasonable request.
